# Special Issue: Advances in Zintl Phases

**DOI:** 10.3390/ma12162554

**Published:** 2019-08-11

**Authors:** Susan M. Kauzlarich

**Affiliations:** Department of Chemistry, One Shields Ave, University of California, Davis, CA 95616, USA; smkauzlarich@ucdavis.edu; Tel.: +1-530-752-4756

**Keywords:** Zintl, intermetallics, structure–property relationships, thermoelectrics, photovoltaics, energy storage, energy conversion

## Abstract

Zintl phases have garnered a great deal of attention for many applications. The term “Zintl phase” recognizes the contributions of the German chemist Eduard Zintl to the field of solid-state chemistry. While Zintl phases were initially defined as a subgroup of intermetallic phases where cations and anions or polyanions in complex intermetallic structures are valence satisfied, the foundational idea of electron counting to understand complex solid-state structures has provided insight into bonding and a bridge between solid-state and molecular chemists. This Special Issue, “Advances in Zintl Phases”, provides a collage of research in the area, from solution to solid-state chemistry.

Zintl phases are a subset of intermetallics where electronic configurations of both cations and anions follow valence rules to ensure stable electronic configurations. The name was initially proposed in recognition of Eduard Zintl’s work on binary phases made from electropositive alkali and alkaline earth metals and main group metalloids, and has remained in place to describe intermetallics that can be considered valence precise. Different from alloys, in which composite elements have no clear oxidation states and electrons are shared by all the elements like metals, Zintl phase compounds are composed of elements of which oxidation states can be clearly calculated and electrons completely transferred from one element to the others. It is conventionally assumed that the electrons are donated from the electropositive metals to the more electronegative elements similar to ionic salt-like compounds, but, in many cases, the total number of electrons is not sufficient to satisfy the octet rule for the more electronegative elements. As a result, the electronegative elements complete a filled valence electron configuration through covalent bonding by making anionic networks. This description considers the electropositive elements as charge-balancing “spectators” that fill the cavities made in the anionic networks. In contrast to this traditional description of Zintl phases, various non-classical Zintl compounds, called polar intermetallic phases, can be formed via integration of transition and rare earth metals, replacing the more electropositive main group and alkali or alkaline earth elements, respectively. These transition metal- or rare earth metal-containing phases can be described as Zintl compounds, showing that the Zintl concept is not limited to elemental compositions with large electronegativity differences. As a result, a wide variety of materials with bonding/electronic structures between that of insulators and metals are possible.

Zintl phases have impacted several areas of research: synthesis of new compounds and crystal structures; new materials via solution chemistry; thermoelectrics; photovoltaics; and energy storage and energy conversion technologies. In addition, some Zintl phases have been identified as topological insulators and Dirac semimetals, materials of importance for their quantum properties. The synthesis of new compounds and crystal structures remains an extremely important research endeavor in the pursuit of technologically relevant materials. The Zintl concept is an excellent pathway to the design of new compounds with exciting technological applications. Using the ideas of Zintl, a specific structure type can be targeted to test ideas for property outcomes and Zintl counting rules can be employed to further optimize an existing structure type; this can provide a foundation for understanding, thereby exploiting structure–property relationships. Additionally, once an understanding of electron counting and bonding is developed, relevant properties can be measured to demonstrate the unique nature of these compounds with an eye towards potential applications. This allows for rational design of new compounds and optimization of existing compounds with an aim towards important technological properties such as thermoelectricity, magnetoelectronics, photovoltaics, and energy storage capabilities.

This Special Issue, “Advances in Zintl Phases”, brings together nine articles and one review, providing a snapshot of the recent activity and development in the field. Zintl phases as reactive precursors are reviewed by Beekman et al. [1]. The potential to tune properties of silicon- and germanium-containing Zintl phases to make novel materials such as clathrates, allotropes, nanoporous and mesostructural materials, nanoparticles, nanosheets, and nanoplates is described. New directions and underexplored areas are highlighted in the review. Gärtner et al. [2] present the synthesis and report on the determination of a tetragonal distortion of NaTl, a phase discovered by Zintl himself, at ambient conditions. Eduard Zintl initiated his investigations of polar intermetallics by reduction of metal halides in sodium-dissolved ammonia solutions. He determined the crystal structure of NaTl from powder diffraction methods as cubic with both sodium and thallium residing in interwoven diamond sublattices. The work by Gärtner et al. is a reinvestigation of Zintl’s original experiment and employed modern powder diffraction methods on phase-pure powders derived from at least two different synthetic methods.

New compounds and new synthetic approaches are the topic of four of the papers. Lithium-containing solid solutions of the polar intermetallic of RE_2_InGe_2_ (RE = La, Nd, Sm, Gd) were investigated by You et al. [3]. These new intermetallics crystallize in the Mo_2_FeB_2_ structure type with Li substituting for In. Employing the Zintl–Klemm formalism for electron counting, two extra valence electrons remained on the metal–metal bond in the conduction band, and the density of states (DOS) calculations implied metallic behavior. Zaikina et al. [4] report the thermal stability and thermoelectric properties of NaZnSb, a new compound prepared using reactive sodium hydride, which is a layered compound isostructural to NaFeAs and LiFeAs superconductors. The thermochemistry was analyzed and thermoelectric properties measured. It is a p-type narrow gap semiconductor with thermal stability up to 700 K. The synthesis of four new phosphide-containing compounds is described by Kovnir et al. [5]. This work demonstrates the richness of Zintl crystal chemistry with a variety of structures varying from zero dimensional (0D), 1D to 2D. The 0D structure of *α*-K_4_P_6_ is reported where P was present as isolated 6 membered P_6_^4−^ rings which lined up but were not bound together. These rings formed nearly flat layers of isolated hexagons with K atoms among the layers. EuP_2_, which is isostructural to SrP_2_ and BaP_2_, formed 1D twisted chains of P. Both *α*- and *β*-EuP_3_ are 2D structural variants. The *α*-form had 14 membered P rings in infinite P layers and the *β*-form contained 22 membered P rings incorporating 6 membered P rings derived from the structure of black P. The thermal stabilities and band gaps were determined. A high-pressure synthesis between 12 and 15 GPa at 800–1050 K used to prepare BaSi_3_, a new distorted variant of the CaGe_3_ structure type, is presented by Schwarz et al. [6]. The crystal structure and chemical bonding properties are presented and reveal covalent bonding in the silicon partial structure and polar multicenter interactions between the silicon layers and the barium atoms. The temperature dependence of the electrical resistivity and magnetic susceptibility were consistent with metallic behavior.

Zintl phases of interest for thermoelectric applications are presented in four publications with new solid solutions and compounds. Kauzlarich et al. [7] present the synthesis, structure, and high-temperature thermoelectric properties of a new solid solution of Yb_14−x_RE_x_ZnSb_11_ (RE = Y, La; x_max_ = 0.5). It shows good efficiency at high temperature with a figure of merit, *zT* ~ 0.7, at 1275 K, attributed to a significant increase in Seebeck with a decreased thermal conductivity. The authors provide a discussion of the complexity of this structure type and show with Pisarenko plots that further optimization of the *zT* is possible. The synthesis and thermoelectric properties of Sc_2_Te_3_ are reported by Bux et al. [8]. Motivated by the high *zT*’s reported for RE_3−x_Te_4_ compounds, this work presents a low-temperature mechanochemical synthesis, high-temperature thermoelectric properties, and thermodynamic parameters such as elastic moduli, speed of sounds, thermal expansion, and Grüneisen parameter. The Sc_2_Te_3_ attained a maximum *zT* value of 0.3 from 500 to 750 K. Several new solid solutions such as Ca_1−x_Eu_x_Cd_2_Sb_2_ and Ca_2−x_Eu_x_CdSb_2_ with the CaAl_2_Si_2_ and Yb_2_CdSb_2_ structure type, respectively, were prepared by Pb flux and described by Bobev et al. [9]. The crystal structure of the solid solution Eu_11−x_Ca_x_Cd_6_Sb_12_ (x ~ 1) is also presented. This work extended the number of solid solutions of the CaAl_2_Si_2_ structure types. The Ca_2−x_Eu_x_CdSb_2_ (x ~ 0.6) crystallized in the Yb_2_CdSb_2_ structure type, whereas the parent Ca_2_CdSb_2_ was its own structure type and Eu_2_CdSb_2_ does not exist. These results suggest a complex dependency of cation size and electronic parameters on structure, as both the Ca_2−x_Eu_x_CdSb_2_ (x ~ 0.6) and Eu_11−x_Ca_x_Cd_6_Sb_12_ (x ~ 1) phases crystallized in structure types where the end members of one side either did not exist or was of a different structure type. While the thermoelectric properties were not measured, these structure types are relevant and worthy of further investigation. The limits of solid solutions in the case of AMg_2_Sb_2_ (A = Mg, Ca, Sr, Ba) alloys are presented by Zevalkink et al. [10]. These phases are of interest for their high thermoelectric figure of merit in the mid-temperature range. The phase stability of the solid solution of Mg with the various cations in the AMg_2_Sb_2_ system, which crystallizes in the CaAl_2_Si_2_ structure type, was investigated. Phase separation was observed and an upper limit for cation radius mismatch in this structure is provided. This work provides general guidance for future alloying and doping studies.
